# Trends and determinants of inequities in childhood stunting in Bangladesh from 1996/7 to 2014

**DOI:** 10.1186/s12939-016-0477-7

**Published:** 2016-11-16

**Authors:** Atonu Rabbani, Akib Khan, Sifat Yusuf, Alayne Adams

**Affiliations:** 1 Department of Economics, University of Dhaka, Dhaka, Bangladesh; 2 James P. Grant School of Public Health, BRAC University, Dhaka, Bangladesh; 3 Health Systems and Population Sciences Division, International Center for Diarrheal Disease Research (icddr,b), Dhaka, Bangladesh

**Keywords:** Stunting, Severe stunting, Socioeconomic inequities, Bangladesh, Bangladesh Demographic and Health Survey, Concentration index, Decomposition

## Abstract

**Background:**

We explore long-term trends and determinants of socioeconomic inequities in chronic childhood undernutrition measured by stunting among under-five children in Bangladesh. Given that one in three children remain stunted in Bangladesh, the socioeconomic mapping of stunting prevalence may be critical in designing public policies and interventions to eradicate childhood undernutrition.

**Methods:**

Six rounds of Bangladesh Demographic and Health Survey data are utilized, spanning the period 1996/97 to 2014. Using recognized measures of absolute and relative inequality (namely, absolute and relative difference, concentration curve and index), we quantify trends, and decompose changes in the concentration index to identify factors that best explain observed dynamics.

**Results:**

Despite remarkable improvements in average nutritional status over the last two decades, socio-economic inequalities have persisted, and according to some measures, even worsened. For example, expressed as rate-ratios, the relative inequality in under-five stunting increased by 56% and the concentration index more than doubled between 1996/97 and 2014. Decomposition analyses find that wealth and maternal factors such as mothers’ schooling and short stature are major contributors to observed socio-economic inequalities in child undernutrition and their changes over time.

**Conclusions:**

Reflecting on recent success around socioeconomic and gender equity in child mortality, and the weak legacy of nutrition policy in Bangladesh, we suggest that nutrition programming energies be focused specifically on the most disadvantaged and applied at scale to close socioeconomic gaps in stunting prevalence.

## Background

Reducing undernutrition among children remains a critical public health and global policy issue. Despite improvements in recent times, globally one in four children of pre-school age remain stunted [13]. Stunting is an anthopometric indicator of chronic undernutrition identifying low height (or length) for age and capturing the cumulative effects of linear growth retardation [48]. At a population level, stunting has been identified as a risk factor for nutrition-related chronic diseases, sub-optimal child growth and development, and reduced health and productivity throughout the life course [12, 42, 45]. Stunting has been found to be responsible for 14.5% of deaths and 12.6% of the total disease burden among under-five children [10], most of which is concentrated in the low and middle-income countries in Africa and south-central Asia.

A substantial literature examines the complex determinants of stunting which include poverty, gender and intra-household biases, low rates of exclusive breastfeeding, inadequate care and complementary feeding, limited access to sanitation facilities, environmental enteropathy and recurrent infections [16, 20, 22, 40, 46]. Understanding socioeconomic specific trends and determinants of stunting are important in designing or targeting interventions that enable greater equity and improved nutrition outcomes.

Over the last three decades, Bangladesh has made significant progress towards reducing under-five stunting. Following the famine of 1974, rates of stunting were reported to be as high as 71% [4]. The current national prevalence of under-five stunting is 36.1%, which represents a 1.2 percentage point decline per annum since 1986 [32]. While this decline is impressive relative to similar or even more advanced developing countries in the region (e.g., India and Pakistan), rates remain unacceptably high. Recent efforts to mainstream priority nutrition interventions into the country’s health system have been criticized for failures in coverage and quality [39]. Given that one in three children remain stunted in Bangladesh, understanding how the prevalence of stunting is distributed across different socio-economic strata may be important in designing public policies and interventions that more effectively target children and families in greatest need [49].

In this context, the present work contributes to a recent body of work that examines stunting trends and determinants. Using data from repeated national surveys in 25 low- and middle-income countries, Restrepo-Méndez et al. [37] have documented a general decline in the prevalence of stunting, accompanied in many cases by worsening equity at the national level. Another strand of literature has employed micro-data for a number of countries, including Bangladesh, to explore associations between under-five stunting and a wide variety of socio-economic and biological factors [15, 16, 26, 36]. Relatively under-researched is the question that links these two literatures: What factors are driving socioeconomic inequities in stunting and their trends?

In this paper, we contribute to this literature by examining trends in stunting over the last 30 years in Bangladesh, with a particular focus on the determinants of stunting inequities and their changes over time. Our exploration of the dynamics and drivers of inequities in stunting may yield insights helpful in strengthening the effectiveness and coverage of priority nutrition interventions both within and beyond the health system. Drawing on six rounds of Bangladesh Demographic and Health Survey (BDHS) data (from 1996/97 to 2014), we use recognized measures of absolute and relative inequality to quantify trends [23], then decompose changes in the concentration index to identify factors that best explain observed dynamics [44].

Key findings are discussed with reference to the history of nutrition policy in Bangladesh, and the experience of large-scale public health interventions that have contributed to pro-poor reductions in childhood mortality [1].

## Methods

### Data

Long term trends in undernutrition among pre-school children of Bangladesh are constructed using five nationally representative sources of data: Child Nutrition Surveys in 1986, 1990, 1992, 1996, 2000, and 2005 [4–9]; Bangladesh Demographic and Health Surveys (BDHS) for the years 1996–97, 1999–00, 2004, 2007, 2011, and 2014 [27–31]; Household Food Security and Nutrition Assessment data for 2009 [47], and annual Bangladesh Food Security and Nutritional Surveillance Project surveys from 2010 to 2013 [17–19]. Together, these sources are used to construct a time-series for under-five stunting and severe stunting spanning a period of almost 30 years since 1986, with cubic spline methods applied to interpolate missing intermediate data [24].

For equity and decomposition analyses we use the last six rounds of the BDHS (1996–97, 1999–00, 2004, 2007, 2011, and 2014). For each round, we measure concentration indices that capture levels and trends in inequities in chronic childhood nutrition. A detailed decomposition exercise is then performed using 1996/97 and 2014 BDHS data that compares the determinants of socioeconomic inequities in stunting and severe stunting over an 18 year period.

### Measurement and analysis

#### Outcome variable: stunting

Undernutrition in children under five most commonly manifests in linear growth failure [35]. This can be captured through the measure of stunting, defined by height less than two standard deviations below the median height for a reference population of a specific age and sex (i.e. height-for-age Z [HAZ] score less than −2). For the purposes of this paper, children who fall below negative two standard deviations (−2 SD) are classified as stunted. We further use −3 standard deviations as a threshold for severe stunting which is associated with a significantly higher risk of long-run cognitive compromise [25].

#### Determinants of socio-economic inequalities in stunting

The literature has identified strong associations between stunting or HAZ scores and a number of socioeconomic and maternal factors [15, 16, 26]. Variables representing these factors are used to explore their contribution to socio-economic inequities in chronic child undernutrition in Bangladesh. Socioeconomic factors are represented by Household Wealth Index, a composite score constructed from the asset vector of the household provided by BDHS based on ownership of selected assets such as televisions and bicycles, materials used for housing construction, and types of water access and sanitation facilities [38]. Maternal factors include years of schooling, chronic energy deficiency [CED] and short stature, along with maternal and child health behaviours such as antenatal doctor visits, birth at a health facility and early breastfeeding. Birth order, age and squared age of the child as well as paternal schooling are also incorporated in the model Wagstaff et al. [44]. We use these variables to explain within-year inequalities in stunting outcomes, as well as between-year changes in equity measures comparing 1996/97 and 2014.

#### Measuring and decomposing socio-economic inequalities

For equity analysis, we start with a simple comparison of stunting/severe stunting rates across different wealth quintiles derived from the BDHS-reported wealth index. Next, we calculate the absolute difference in under-five undernutrition prevalence between the richest and poorest wealth quintiles to capture the size of the disparity between the two groups. We also report the ratio of under-five stunting/severe stunting rates between these two cohorts to gauge the relative disadvantage of the poorest group in comparison to the richest one. While absolute and relative differences between the richest and the poorest groups are common equity indicators, these measures do not exploit the entire distribution of health outcomes over relevant socio-economic characteristics at the household level. Such measures can also be prone to extreme values. Hence, we construct concentration curves which plot the cumulative share of under-five stunting against the cumulative percentage of the population ranked from the poorest to the richest [23]. We also calculate the ‘concentration index’ (C) which summarizes information contained in each concentration curve [33]. Quantitatively, the concentration index is twice the area between the concentration curve and the line of equality (or the 45° line, which represents a perfectly equal distribution of a health outcome; see Fig. 4). The index ranges from −1 to 1. C is negative when the curve lies above the 45° line, indicating a higher incidence of stunting among the poor (a value of 0 signifies perfect equality). The higher the relative burden of stunting borne by the poorer cohorts, the further away and above the curve is with respect to the line of equality and the closer the index is to −1. Given that the dichotomous nature of our outcome variable may pose a problem in the form of varying bounds of the index in response to changing means, we further normalize the standard index estimates to check for robustness [43]. We make use of both concentration curves and indices to track the dynamics of socio-economic differences in under-five stunting and severe stunting. Statistical tests of dominance between concentration curves are carried out to evaluate differences in stunting inequity across time.

In addition, using the methods outlined in Wagstaff et al. [44] and Oaxaca [34], we decompose socio-economic inequity in stunting to identify the contribution of different explanatory factors comparing 1996/97 and 2014. Combining the concentration index with regression analyses, these methods help us explore the causes of (and their relative contributions to) levels of and changes in stunting inequities. To elaborate, Wagstaff et al. [44], show that the contribution of a factor to the concentration index of a health outcome is essentially the product of its own concentration index and the elasticity of health outcome with respect to that factor. Consequently, changes in the concentration index over time can be decomposed into changes due to: a) changing concentration index of a factor, and b) changing elasticity of outcome with respect to that factor. We use Stata 13® for statistical analyses. All the estimates take sampling weights into consideration.

## Results

### Long-term trends in chronic undernutrition

**Fig. 1:**
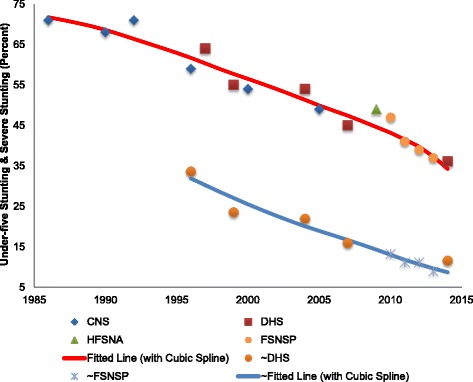
Long-term Trends in Under-five Stunting (Height-for-age z score < −2) & Severe Stunting (Height-for-age z score < −3). Note: ‘~’ signifies estimates of severe stunting rates

Figure 1 presents long-term trends in under-five stunting in Bangladesh over a period of almost three decades between 1986 and 2014. In the mid-1980s, according to the available data from the *Child Nutrition Survey*, about 71% of children under five were stunted. This high rate of stunting persisted until the early 1990s, followed by a period of decline that averaged 1.6 percentage points (or 2.2%) per annum, which represents or a 50% drop in the span of 22 years. Figure 1 also suggests a continuous fall in severe stunting among pre-school children – albeit at a somewhat lower absolute rate than that for stunting - from 34% in 1996/97 to about 11.6% in 2014.

### Socio-economic inequalities in stunting and severe stunting

**Fig. 2:**
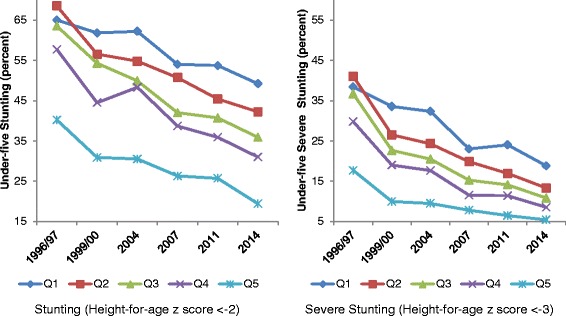
Trends in Stunting and Severe Stunting Across Wealth Quintiles. Note: Estimates are based on BDHS data. Qi = i-th wealth quintile (i = 1, 2,..,5; higher i represents a quintile with higher wealth)

Figure 2 depicts trends in stunting and severe stunting stratified by wealth quintiles using data from six consecutive rounds of BDHS. While the rate of stunting consistently declined across all five wealth quintiles comparing 2014 and 1996/97, the reduction was relatively greater among the richest quintile compared to the poorest. In 1996/97, stunting prevalence was about 65% in the lowest wealth quintile and about 40% in the highest one. By 2014, these dropped to about 49 and 19%, respectively. The average annual decline during this period was 1.5 percentage points for the richest cohort as opposed to about 1.1 percentage points for the poorest (see Table 1). Interestingly, the intermediate wealth groups posted considerably higher rates of reduction of about two percentage points. For severe stunting, however, prevalence trended downward across all strata with the poorest quintile performing better than their richest counterpart in terms of annualized absolute decline (1.4 compared to 0.9 percentage points). Like stunting, this rate was higher for the cohorts in between. On the other hand, if we consider annualized relative change i.e. *percent* decline per annum, there was a strong positive association between improvement in under-five stunting and household wealth, suggesting that improvements in childhood nutrition accrued disproportionately to more affluent households.

**Table 1:** Wealth-stratified Long-term Rates of Decline in Child Undernutrition in Bangladesh

Wealth Quintile	1996/97 (percent)	2014 (percent)	Time Difference (2) – (1) (percentage points)	Annualized Absolute Decline (percentage points per annum)	Annualized Relative Decline (percent per annum)
	(1)	(2)			
Stunting					
Q1	65	49	16	1.1	2.0
Q2	69	42	27	1.9	3.5
Q3	64	36	28	2.0	4.1
Q4	58	31	27	1.9	4.5
Q5	40	19	21	1.5	5.2
Severe Stunting					
Q1	38	19	20	1.4	5.1
Q2	41	13	28	2.0	8.0
Q3	37	11	26	1.8	8.7
Q4	30	9	21	1.5	9.0
Q5	18	5	12	0.9	8.5

Note: Estimates are based on BDHS data. Q_i_ = i-th wealth quintile (i = 1, 2,..,5; higher i represents a quintile with higher wealth)

**Fig. 3:**
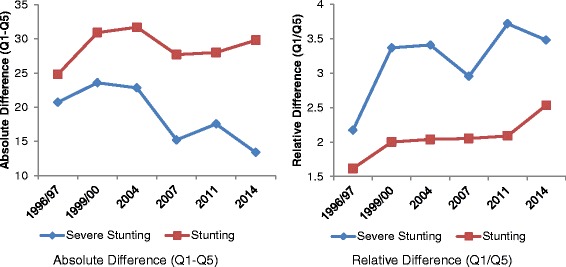
Dynamics of Stunting and Severe Stunting Inequality. Note: Estimates are based on BDHS data. Qi = i-th wealth quintile; Q1 (5) represents the poorest (richest) 20%

Measures of inter-quintile differences provide further support to initial observations of sustained and sometimes worsening socio-economic inequities in under-five stunting between 1996/97 and 2014. The absolute difference in stunting rates between the top and bottom wealth quintiles increased from 25 to about 30 percentage points between 1996/97 and 2014 (see Table 1 and Fig. 3), suggesting a worsening of social inequities that were very high to start with. The corresponding trend for severe stunting was less definitive, but still indicative of persistent inequities. In 2014, there was a 13.4 percentage points difference between the two extreme socioeconomic quintiles. Measures of relative inequity showed substantial increases over time for both stunting and severe stunting. Expressed as a rate-ratio comparing rates of stunting between the poorest and richest quintiles, relative inequity was 1.6 in 1996/97 and increased to 2.5 in 2014 (a 56% increase; see Fig. 3). Expressed in terms of probability, in 2014, a child from the poorest quintile was more than twice as likely to fail to reach his/her growth potential compared to a child from the richest quintile. The rate ratio for severe stunting rose even more, by almost 59%, from 2.2 to 3.5. These figures are consistent with the variation in annual declines in stunting between different socioeconomic groups (as reported in Table 1). So, while the overall prevalence has fallen in recent times, children from the lower wealth quintiles are bearing an increasing share of burden of stunting compared to those from the higher wealth quintiles.

**Fig. 4:**
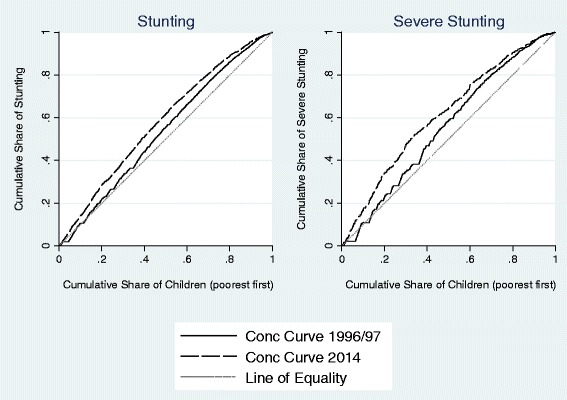
Concentration Curves for Stunting and Severe Stunting, 1996/97 and 2014. Note: Estimates are based on BDHS data

Trends in equity measures that take into consideration variations in nutritional outcomes across the entire wealth distribution provide additional support to these findings. Figure 4 presents concentration curves for stunting and severe stunting in 1996/97 and 2014. In 1996/97, for both indicators of chronic child undernutrition, the concentration curve was consistently above the so-called line of equality, suggesting a disproportionate concentration of growth faltering among poorer households. By 2014, the curves shifted even further away, indicating a rise in the degree of inequality such that the poorest 40% accounted for more than half of all stunted children. Deepening inequities between rich and poor quintiles are even more apparent for severe stunting, with the bottom third of the distribution bearing half of the burden. In both cases, we can reject the null of non-dominance at the 1% level of significance using the test outlined in O’Donnell and Wagstaff [33].

**Fig. 5:**
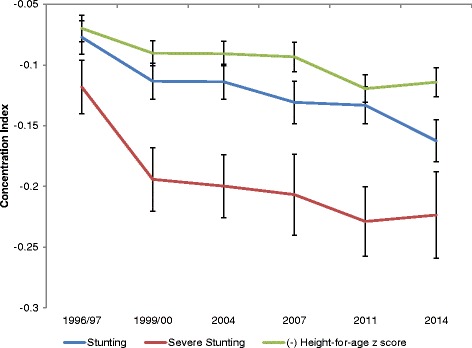
Dynamics of Concentration Index of Stunting & Severe Stunting. Note: Estimates are based on BDHS data

The estimated concentration indexes for all time periods are presented in Fig. 5 (and further detailed in Table 2). Consistent with the results above, the concentration index for under five stunting significantly increased (in absolute value) from 0.077 in 1996/97 to 0.162 in 2014. The negative values indicate that stunting was disproportionately concentrated in poorer households. The same was true for severe stunting. However, index values for severe stunting were significantly higher (in absolute value) than those for stunting. In short, socio-economic inequities in stunting, as measured by concentration index, persisted if not worsened during the period under study. This conclusion does not change if we consider only ‘normalized’ concentration index estimates. Moreover, if we look at the index estimates for the negative of HAZ scores as an alternative, we find even stronger evidence that the burden of child undernutrition became more concentrated among the poorer sections of the population between 1996/97 and 2014.

**Table 2:** Trends in the Concentration Index of Child Undernutrition

BDHS	Rate (percent)/Mean	Concentration Index (C)	Standard Error (of C)	95% Confidence Interval		Normalized Conc. Index
				Lower	Upper	
Stunting						
1996/97	60.0	−0.077	0.007	−0.091	−0.063	−0.193
1999/00	51.2	−0.113	0.008	−0.128	−0.098	−0.232
2004	50.5	−0.114	0.007	−0.128	−0.099	−0.230
2007	43.0	−0.131	0.009	−0.148	−0.113	−0.229
2011	41.2	−0.133	0.008	−0.148	−0.118	−0.226
2014	36.1	−0.162	0.009	−0.180	−0.145	−0.254
Severe Stunting						
1996/97	33.6	−0.118	0.011	−0.140	−0.096	−0.178
1999/00	23.5	−0.194	0.013	−0.220	−0.168	−0.254
2004	21.9	−0.200	0.013	−0.226	−0.174	−0.256
2007	15.9	−0.207	0.017	−0.240	−0.173	−0.246
2011	15.2	−0.229	0.015	−0.258	−0.200	−0.270
2014	11.6	−0.224	0.018	−0.259	−0.188	−0.253
(Negative) Height-for-age z score						
1996/97	2.33	−0.070	0.006	−0.081	−0.059	-
1999/00	2.03	−0.090	0.005	−0.101	−0.080	-
2004	1.98	−0.091	0.005	−0.101	−0.081	-
2007	1.75	−0.093	0.006	−0.105	−0.081	-
2011	1.67	−0.119	0.006	−0.131	−0.108	-
2014	1.54	−0.114	0.006	−0.126	−0.102	-

Note: Estimates are based on BDHS data. Normalization of C involves dividing the concentration index by (1-rate)

### Decomposition of temporal and inter-temporal stunting inequalities

Three sets of variables are consistently associated with stunting outcomes in both 1996/97 and 2014 (please see Table 3). Firstly, age of the child exhibits a hump-shaped association with stunting outcomes: Ceteris paribus, the probability of stunting increases with age until 38 months, after which it starts to diminish. Secondly, mother’s education level and physical stature are statistically significant determinants for stunting. Children of more educated mothers are less prone to be stunted, as are those with taller mothers, which is suggestive of an intergenerational link in child nutritional outcomes. Furthermore, a comparatively higher elasticity for mother’s education in 2014 suggests that maternal education has become more important in determining children’s nutritional health than 18 years earlier. A final variable that is consistent in explaining stunting outcomes is household wealth. Children in richer households have a lower probability of being stunted. Moreover, a higher elasticity of household wealth in 2014 compared to 1996/97 (in absolute value) suggests that this factor has become more important in determining under-five stunting over time. Among other variables, paternal education was a significant determinant in 1996/97 but not in 2014, with a decline in elasticity as well. Delivery at a health facility became significant and also registered a higher elasticity in 2014.

**Table 3:** Oaxaca Decomposition for Changes in Stunting Inequality between 1996/97 & 2014

Variables	E: Elasticity C: Conc. Index t:2014 (t-1): 1996/97					
	Variation (1)		Variation (2)			
	E_t_(C_t_-C_t-1_)	C_t-1_(E_t_-E_t-1_)	E_t-1_(C_t_-C_t-1_)	C_t_(E_t_-E_t-1_)	Total	%
Child’s age	−0.007	−0.007	−0.004	−0.010	−0.014	16.66
Child’s age squared	0.019	0.006	0.007	0.018	0.025	−29.79
Birth Order	0.002	0.002	−0.001	0.005	0.004	−4.52
Antenatal visit to doctor	0.007	−0.009	0.003	−0.005	−0.002	2.63
Delivery at health facility	0.013	−0.022	0.001	−0.010	−0.009	10.58
Early initiation of breastfeeding	−0.004	0.003	0.002	−0.003	−0.001	0.68
Maternal Schooling (years)	0.038	−0.052	0.010	−0.024	−0.015	17.16
Paternal Schooling (years)	0.003	0.007	0.004	0.005	0.009	−11.13
Maternal CED (Chronic Energy Deficiency)	−0.003	0.000	−0.002	−0.001	−0.003	4.03
Maternal short stature (<145 cm)	−0.007	−0.001	−0.005	−0.002	−0.008	8.98
Wealth Index	−0.013	−0.009	−0.009	−0.013	−0.022	25.82
Residual					−0.050	58.89
Total	0.047	−0.082	0.006	−0.041	−0.085	

Note: Variation 1 (2) uses E_t_ (E_t-1_) and C_t-1_ (C_t_) to weight changes in C and E respectively. See Wagstaff et al. [44] for details

Comparing 1996/97 and 2014, variables that significantly contribute to stunting inequities across surveys include household wealth as well as mother-specific factors such as schooling and sub-optimal stature. Collectively, they accounted for more than 50% of the concentration index estimates.

We further decomposed the aggregate change in concentration index (of stunting) between 1996/97 and 2014. The results are reported in Table 3. Once again, household wealth emerges as the most important factor explaining the increase in stunting inequity. Not only has wealth inequity increased between the two time periods, the sensitivity of under-five stunting to household wealth has also increased as suggested by a higher level of elasticity per Table 4. As a result, about 26% of the change in concentration index (C) can be attributed to wealth level. The second most important explanatory factor is maternal education contributing about 17% of the observed change in C. While the distribution of maternal schooling has become less inequitable over time (Table 4), it has been offset by increased sensitivity of childhood stunting to maternal education apparent in its substantial contribution (17%) to the rise in C. Maternal physical stature also contributed about 9% to the increase in C through a rise in both sensitivity and its own inequality. However, the very large residual in this analysis suggests there are other unobserved factors underlying the increase in C of under-five stunting.^1^

**Table 4:** Decomposition of the Concentration Index for Stunting (1996/97 & 2014)

Variables	Coefficients & p-values				Elasticities		Concentration Index (C)		Contributions to C (%)		
	1996/97		2014		1996/97	2014	1996/97	2014	1996/97	2014	Change
Child’s age	0.03	0.00	0.03	0.00	1.30	2.48	−0.006	−0.009	9.58	13.30	3.71
Child’s age squared	0.00	0.00	0.00	0.00	−0.68	−1.82	−0.005	−0.016	−4.82	−17.92	−13.10
Birth Order	0.00	0.35	−0.01	0.28	0.02	−0.04	−0.042	−0.083	1.02	−1.89	−2.90
Antenatal visit to doctor	−0.05	0.02	−0.03	0.23	−0.02	−0.04	0.363	0.199	8.14	5.25	−2.89
Delivery at health facility	−0.05	0.16	−0.04	0.05	0.00	−0.04	0.638	0.302	3.09	7.02	3.93
Early initiation of breastfeeding	−0.07	0.00	0.02	0.19	−0.02	0.03	0.062	−0.055	1.67	1.15	−0.52
Maternal Schooling (years)	−0.01	0.00	−0.01	0.00	−0.04	−0.17	0.409	0.188	22.81	19.85	−2.97
Paternal Schooling (years)	−0.01	0.00	0.00	0.35	−0.05	−0.03	0.343	0.260	23.53	5.34	−18.19
Maternal CED (Chronic Energy Deficiency)	0.02	0.27	0.03	0.10	0.02	0.02	−0.101	−0.246	2.10	3.11	1.02
Maternal short stature (<145 cm)	0.17	0.00	0.19	0.00	0.05	0.07	−0.043	−0.149	2.79	6.04	3.25
Wealth Index	−0.04	0.00	−0.03	0.03	−0.29	−0.41	0.074	0.105	27.19	26.47	−0.72
Total									97.11	67.72	−29.38

Note: *N* = 3288 for 1996/97 and 3884 for 2014. All regressions are probit. Coefficients are average marginal effects. Standard errors used take into account sampling weights. In addition to the variables reported, all regressions control for division-specific fixed effects. Antenatal visit to doctor, delivery at health facility, early breastfeeding, and maternal CED and short stature are all dummy variables

Decomposition analysis for severe stunting yields somewhat similar results (see Tables 5 and 6). While maternal schooling and short stature continue to be significant contributors to the levels of and change in severe stunting inequities among under-fives, household wealth ceases to be an explanatory factor in 2014. Rather, prenatal doctor visit emerges as important, accounting for almost 12% of the increase in the severe stunting concentration index due to a remarkable increase in elasticity.^2^

**Table 5:** Decomposition of the Concentration Index for Severe Stunting (1996/97 & 2014)

Variables	Coefficients & p-values				Elasticities		Concentration Index (C)		Contributions to C (%)		
	1996/97		2014		1996/97	2014	1996/97	2014	1996/97	2014	Change
Child’s age	0.022	0.00	0.014	0.00	1.88	3.47	−0.006	−0.009	9.08	13.54	4.46
Child’s age squared	0.000	0.00	0.000	0.00	−1.01	−2.81	−0.005	−0.016	−4.71	−20.07	−15.37
Birth Order	0.002	0.56	0.005	0.20	0.02	0.09	−0.042	−0.083	0.70	3.44	2.74
Antenatal visit to doctor	−0.041	0.06	−0.021	0.08	−0.02	−0.11	0.363	0.199	7.44	9.40	1.97
Delivery at health facility	0.067	0.11	−0.022	0.10	0.01	−0.07	0.639	0.302	−4.40	9.79	14.20
Early initiation of breastfeeding	−0.033	0.13	0.007	0.53	−0.02	0.03	0.062	−0.055	0.90	0.73	−0.17
Maternal Schooling (years)	−0.012	0.00	−0.006	0.00	−0.08	−0.32	0.408	0.188	28.28	26.99	−1.29
Paternal Schooling (years)	−0.010	0.00	−0.001	0.65	−0.10	−0.04	0.343	0.260	28.77	4.97	−23.80
Maternal CED (Chronic Energy Deficiency)	0.047	0.00	0.010	0.47	0.07	0.02	−0.101	−0.246	6.18	2.15	−4.03
Maternal short stature (<145 cm)	0.122	0.00	0.078	0.00	0.07	0.09	−0.041	−0.149	2.33	5.82	3.50
Wealth Index	−0.035	0.00	−0.011	0.26	−0.51	−0.47	0.074	0.105	32.01	22.13	−9.88
Total									106.58	78.89	−27.69

Note: *N* = 3288 for 1996/97 and 3884 for 2014. All regressions are probit. Coefficients are average marginal effects. Standard errors used take into account sampling weights. In addition to the variables reported, all regressions control for division-specific fixed effects. Antenatal visit to doctor, delivery at health facility, early breastfeeding, and maternal CED and short stature are all dummy variables

**Table 6:** Oaxaca Decomposition for Changes in Severe Stunting Inequality between 1996/97 & 2014

Variables	E: Elasticity C: Conc. Index t:2014 (t-1): 1996/97					
	Variation (1)		Variation (2)			
	E_t_(C_t_-C_t-1_)	C_t-1_(E_t_-E_t-1_)	E_t-1_(C_t_-C_t-1_)	C_t_(E_t_-E_t-1_)	Total	%
Child’s age	−0.010	−0.009	−0.006	−0.014	−0.020	18.53
Child’s age squared	0.029	0.010	0.011	0.029	0.039	−37.29
Birth Order	−0.004	−0.003	−0.001	−0.006	−0.007	6.50
Antenatal visit to doctor	0.017	−0.030	0.004	−0.016	−0.012	11.60
Delivery at health facility	0.024	−0.051	−0.003	−0.024	−0.027	25.70
Early initiation of breastfeeding	−0.003	0.003	0.002	−0.003	−0.001	0.54
Maternal Schooling (years)	0.071	−0.098	0.018	−0.045	−0.027	25.54
Paternal Schooling (years)	0.004	0.019	0.008	0.015	0.023	−21.70
Maternal CED (Chronic Energy Deficiency)	−0.003	0.005	−0.010	0.013	0.002	−2.36
Maternal short stature (<145 cm)	−0.009	−0.001	−0.007	−0.003	−0.010	9.74
Wealth Index	−0.015	0.003	−0.016	0.005	−0.012	11.06
Residual					−0.055	52.13
Total	0.101	−0.151	0.000	−0.050	−0.105	

Note: Variation 1 (2) uses E_t_ (E_t-1_) and C_t-1_ (C_t_) to weight changes in C and E respectively. See Wagstaff et al. [44] for details

## Discussion

Bangladesh is widely celebrated for achieving substantial and rapid population health gains over the last three decades [11]. Among these acheivements are overall improvements in child survival which have occurred in a short span of time and with notable equity gains [1]. Our analysis reveals a similar reduction in chronic child undernutrition since the late 1980s when more than two-thirds of the under-five children were short for their age. Remarkably, rates of stunting in Bangladesh are currently lower than neighboring India and Pakistan where per-capita incomes are substantially higher than Bangladesh [11]. Even then, the current prevalence of stunting still remains high, affecting 35% of under-five children in 2014.

Our analysis also suggests increasing socio-economic inequalities in stunting over time. Based on the analysis of BDHS data over a period spanning 18 years, we observed a decline in under-five stunting and severe stunting across the socio-economic spectrum, but with the rate of improvement in the richest quintile significantly outpacing that of the poorest. Consequently, socioeconomic disparities persisted and according to some of our estimates, worsened over time. By 2014, the difference in the prevalence of stunting comparing rich and poor quintiles increased to 30 percentage points (a 2.5 rate ratio). According to concentration curve and index analyses, pro-rich improvements were even more pronounced in the distribution of severe stunting. Similar trends indicating decreasing rates of stunting, accompanied by increasing socio-economic inequities have been noted in the region. In India, during the period 1993–2006, stunting declined at a rate of 42% in the richest quintile and only 14 in the poorest [41]. Stunting also fell in Nepal (Cambodia) between 2001 and 2011 (2000 and 2010) by 17 (10.5) percentage points, but the rate of reduction was significantly higher in the richest quintile compared to the poorest one - 41 vs. 17% (31 vs. 15) [37].

The results of decomposition analyses go even further, suggesting that socioeconomic inequalities are the most important factor driving inequities in under-five stunting. From a public health perspective, policy efforts that tackle structural factors such as unequal wealth distribution including social safety nets, employment creation and fair taxation, may help address inequities in stunting. Decomposition analysis also reveals that mother’s education and physical stature are critical in explaining levels of and changes in socio-economic inequities in stunting from 1996/97 to 2014. The strong intergenerational nature of stunting emphasizes the need for interventions that break the cycle of transmission or enable escape from the so-called nutrition-based poverty traps. Here, nutrition-specific interventions targeting low income households and communities may be particularly important, such as appropriate infant and young child feeding during the first 2 years of life, micronutrient supplementation, and improved food and nutrient intake among adolescent girls and women, especially during pregnancy. In our analysis, prenatal visits to a doctor, a key component of maternal care, was also an important determinant of socioeconomic inequalities in stunting. Among the severely stunted children, this was particularly apparent, emphasizing the priority that prenatal care should receive in efforts to improve fetal growth, and ultimately, to reduce levels and disparities in severe stunting.

It is perplexing why declines in stunting lack the pro-poor trends observed for under-five mortality over a similar time period [1]. While it might be assumed that increasing inequities in maternal nutritional status and household wealth would exacerbate inequities in both child survival and nutrition outcomes, this was not reflected in trends in child mortality where pro-poor improvements have been documented.

A potential explanation may lie in the differential policy environments associated with nutrition and health interventions. As Adams et al. [1] note, large-scale pro-poor interventions such as the Expanded Program on Immunization and the popularization of Oral Rehydration Therapy were important drivers of equity gains in child survival. The short history of nutrition policy and programming, however, appears to lack the scale and strong public and political commitment associated with successful community-based public health interventions.

Bangladesh has adopted numerous policies over the years to address food insecurity and malnutrition, the most ambitious of which was the National Nutrition Program (NNP). Initiated in 1997, and implemented alongside health and related sectors in a parallel fashion, NNP focused primarily on nutrition-specific interventions. With reported coverage rates of only 30% of the total population [2], concerns were raised about the vertical nature of NNP, and the lack of institutional mechanisms and resources to engage other sectors in critical nutrition-sensitive programming. Due to poor performance in the initial years of the program, and the need to place greater emphasis on improving coverage and efficiency, the National Nutrition Services (NNS) was created in 2011 to steward nutrition mainstreaming into health and related sectors. Implementation, however, has been slow due to poor management and coordination at the district and sub-district (upazila) levels, and the lack of skilled health workers able to deliver an ambitious range of nutrition services [14, 39]. The recent deployment of District Nutrition Support Officers to troubleshoot and support effective implementation and results-based training for health workers are efforts to overcome these challenges [21]. Despite these efforts, rates of Vitamin A supplementation among children aged 9–59 months have declined from 80% in 1999 to 63% in 2014 and the percentage of children aged 6–23 months fed according to recommended infant and child feeding practices increased only 2% from 21% in 2011 to 23% in 2014 [28, 31, 32]. Similarly, the national deworming program catered to only three-fourth of its under-specified target population in 2013 (children aged 24–59 months although WHO recommends 12–59 months) [19]. Issues of coverage appear to be of urgent concern, and calls for greater focus on the quality delivery of priority interventions and more effective outreach, appear consistent with our findings [39]. In this context, overcoming geographic inequities in service provision, and targeting the poorest who represent the group least likely to access or avail services, are important policy priorities.

Our findings also support important ethical arguments for the design of targeted policy and programs. Absolute declines in under-five stunting across all wealth groups suggest that improvement in nutritional status has not come at the cost of any particular wealth group, implying a Pareto improvement. At the same time, our findings indicate that relative inequalities have increased significantly over the study period, and suggest a deteriorating scenario in under-five stunting outcomes in strict egalitarian terms [3]. Even if we take a more balanced approach towards measuring inequality with disproportionately more weight on shortfalls in childhood under-nutrition among the lower wealth groups, as concentration indexes allow, we find evidence that inequity has worsened for the households from the poorer strata and gains have accrued disproportionately to the higher wealth groups. These findings are important to consider in the design of policies and interventions to improve under-five nutrition, and suggest that priority efforts be focused on lower wealth groups.

## Conclusions

While the broader determinants of inequities in stunting are important to address, including the promotion of inter-sectoral nutrition-sensitive strategies in the areas of agriculture and food security, social protection, education, water and sanitation, and health and family planning services, concerted efforts towards increasing effective coverage of nutrition-specific interventions are critical if equity is to be improved. Learning from the example of Bangladesh’s success around socioeconomic and gender equity in child mortality, more targeted approaches may be required which focus specifically on high risk groups, and which are applied at scale. Of particular concern is the first 1000 days from conception to the second year of a child’s life, during which children’s linear growth and development are most sensitive to feeding and care [41]. Here, redoubled efforts around exclusive breastfeeding for the first 6 months of a child’s life, timely introduction of hygienic and age-appropriate complementary foods in terms of frequency, density and diversity, and careful management of diet and therapeutic treatment in cases of illness or severe wasting are needed. According to the 2014 BDHS, 64% of children were fed with minimum meal frequency, 23% with a minimally acceptable diet, and only 28% with a minimum dietary diversity (National Institute of Population Research and Training et al. [32]). Greater priority should also be given to the health and nutrition of female adolescents and those from the poorest socioeconomic quintiles, in order to break the intergenerational cycle of stunting, and improve women’s pregnancy and lactation outcomes. Here, nutrition-specific Interventions such as adolescent health and preconception nutrition, and programs focused on improving the quality and quantity of maternal diet, and dietary supplementation, are critical. Focusing nutrition programming energies more specifically, and addressing socioeconomic inequities deliberately through a focus on the most disadvantaged, will contribute to decreased stunting at the population level.

## Appendix

**Table 7:** Decomposition of the Concentration Index for Negative Height-for-age Z scores (1996/97 & 2014)

Variables	Coefficients & p-values				Elasticities		Concentration Index (C)		Contributions to C (%)		
	1996/97		2014		1996/97	2014	1996/97	2014	1996/97	2014	Change
Child’s age	0.097	0.00	0.112	0.00	1.21	2.13	−0.006	−0.009	10.11	16.20	6.09
Child’s age squared	−0.001	0.00	−0.002	0.00	−0.63	−1.53	−0.006	−0.016	−5.10	−21.35	−16.25
Birth Order	0.001	0.94	0.007	0.72	0.00	0.01	−0.042	−0.083	0.08	0.69	0.62
Antenatal visit to doctor	−0.129	0.06	−0.144	0.00	−0.01	−0.05	0.364	0.199	5.66	9.42	3.76
Delivery at health facility	0.068	0.55	−0.082	0.09	0.00	−0.02	0.639	0.302	−1.09	5.32	6.41
Early initiation of breastfeeding	−0.100	0.12	0.085	0.07	−0.01	0.03	0.062	−0.055	0.67	1.39	0.73
Maternal Schooling (years)	−0.041	0.00	−0.025	0.02	−0.04	−0.10	0.408	0.188	23.28	15.79	−7.49
Paternal Schooling (years)	−0.029	0.00	−0.012	0.11	−0.04	−0.04	0.343	0.260	20.27	9.73	−10.54
Maternal CED (Chronic Energy Deficiency)	0.162	0.00	0.126	0.02	0.04	0.02	−0.101	−0.246	5.18	3.94	−1.25
Maternal short stature (<145 cm)	0.543	0.00	0.649	0.00	0.04	0.05	−0.042	−0.150	2.59	7.04	4.45
Wealth Index	−0.163	0.00	−0.120	0.00	−0.34	−0.39	0.074	0.105	36.07	35.48	−0.59
Total									97.71	83.64	−14.08

Note: *N* = 3288 for 1996/97 and 3884 for 2014. OLS regression. Standard errors used take into account sampling weights. In addition to the variables reported, all regressions control for division-specific fixed effects. Antenatal visit to doctor, delivery at health facility, early breastfeeding, and maternal CED and short stature are all dummy variables

**Table 8:** Oaxaca Decomposition for Changes in Negative HAZ score Inequality between 1996/97 & 2014

Variables	E: Elasticity C: Conc. Index t: 2014 (t-1): 1996/97					
	Variation (1)		Variation (2)			
	E_t_(C_t_-C_t-1_)	C_t-1_(E_t_-E_t-1_)	E_t-1_(C_t_-C_t-1_)	C_t_(E_t_-E_t-1_)	Total	%
Child’s age	−0.006	−0.005	−0.003	−0.008	−0.011	25.81
Child’s age squared	0.016	0.005	0.006	0.014	0.021	−47.00
Birth Order	0.000	0.000	0.000	−0.001	−0.001	1.67
Antenatal visit to doctor	0.009	−0.016	0.002	−0.009	−0.007	15.34
Delivery at health facility	0.007	−0.014	0.000	−0.006	−0.007	15.43
Early initiation of breastfeeding	−0.003	0.002	0.001	−0.002	−0.001	2.54
Maternal Schooling (years)	0.021	−0.023	0.009	−0.011	−0.002	3.97
Paternal Schooling (years)	0.004	0.000	0.003	0.000	0.003	−6.91
Maternal CED (Chronic Energy Deficiency)	−0.003	0.002	−0.005	0.004	−0.001	1.97
Maternal short stature (<145 cm)	−0.006	0.000	−0.005	−0.002	−0.006	14.06
Wealth Index	−0.012	−0.003	−0.011	−0.005	−0.015	34.55
Residual					−0.017	38.57
Total	0.026	−0.053	−0.003	−0.024	−0.044	

Note: Variation 1 (2) uses E_t_ (E_t-1_) and C_t-1_ (C_t_) to weight changes in C and E respectively. See Wagstaff et al. [44] for details

## Abbreviations

BDHS: Bangladesh Demographic and Health Survey
C: Concentration index
CED: Chronic energy deficiency
HAZ: Height-for-age Z
NNP: National nutrition program
SD: Standard deviation

## Footnotes

1 The conclusions do not change remarkably when we consider negative height-for-age z scores as the outcome variables. The results are reported in Tables 7 and 8 in Appendix.

2 Statistically, the variables included in our model can only capture observed variation generating the inequality in the stunting outcomes. We have included the determinants as allowed by the data we are using (DHS) and also guided by the prior literature (see Wagstaff et al. [44], Headey et al. [16]). It is interesting that the typical set of explanatory variables used to explain inequality in stunting outcomes had less statistical power in 2014, and suggests the need for renewed thinking about best policy approaches for redressing inequities.
