# Erratum to: Trends and determinants of inequities in childhood stunting in Bangladesh from 1996/7 to 2014

**DOI:** 10.1186/s12939-016-0495-5

**Published:** 2017-01-04

**Authors:** Atonu Rabbani, Akib Khan, Sifat Yusuf, Alayne Adams

**Affiliations:** 1 Department of Economics, University of Dhaka, Dhaka, Bangladesh; 2 James P. Grant School of Public Health, BRAC University, Dhaka, Bangladesh; 3 Health Systems and Population Sciences Division, International Center for Diarrheal Disease Research (icddr,b), Dhaka, Bangladesh

## Erratum

Unfortunately, after publication of this article [1], it was noticed that Tables 3 and 4 were inadvertently swapped during the production process. The tables with their correct table citation can be seen below and the original article has been update to reflect this.

**Table 3:** Decomposition of the concentration index for stunting (1996/97 & 2014)

	Coefficients & *p*-values				Elasticities		Concentration index (C)		Contributions to C (%)		
Variables	1996/97		2014		1996/97	2014	1996/97	2014	1996/97	2014	Change
Child's age	0.03	0.00	0.03	0.00	1.30	2.48	−0.006	−0.009	9.58	13.30	3.71
Child's age squared	0.00	0.00	0.00	0.00	−0.68	−1.82	−0.005	−0.016	−4.82	−17.92	−13.10
Birth Order	0.00	0.35	−0.01	0.28	0.02	−0.04	−0.042	−0.083	1.02	−1.89	−2.90
Antenatal visit to doctor	−0.05	0.02	−0.03	0.23	−0.02	−0.04	0.363	0.199	8.14	5.25	−2.89
Delivery at health facility	−0.05	0.16	−0.04	0.05	0.00	−0.04	0.638	0.302	3.09	7.02	3.93
Early initiation of breastfeeding	−0.07	0.00	0.02	0.19	−0.02	0.03	0.062	−0.055	1.67	1.15	−0.52
Maternal Schooling (years)	−0.01	0.00	−0.01	0.00	−0.04	−0.17	0.409	0.188	22.81	19.85	−2.97
Paternal Schooling (years)	−0.01	0.00	0.00	0.35	−0.05	−0.03	0.343	0.260	23.53	5.34	−18.19
Maternal CED (Chronic Energy Deficiency)	0.02	0.27	0.03	0.10	0.02	0.02	−0.101	−0.246	2.10	3.11	1.02
Maternal short stature (<145 cm)	0.17	0.00	0.19	0.00	0.05	0.07	−0.043	−0.149	2.79	6.04	3.25
Wealth Index	−0.04	0.00	−0.03	0.03	−0.29	−0.41	0.074	0.105	27.19	26.47	−0.72
Total									97.11	67.72	−29.38

Note: *N* = 3,288 for 1996/97 and 3,884 for 2014. All regressions are probit. Coefficients are average marginal effects. Standard errors used take into account sampling weights. In addition to the variables reported, all regressions control for division-specific fixed effects. Antenatal visit to doctor, delivery at health facility, early breastfeeding, and maternal CED and short stature are all dummy variables

**Table 4:** Oaxaca decomposition for changes in stunting inequality between 1996/97 & 2014

	E: Elasticity C: Conc. Index t:2014 (t-1): 1996/97					
	Variation (1)		Variation (2)			
Variables	E_t_ (C_t_-C_t-1_)	C_t-1_ (E_t_-E_t-1_)	E_t-1_ (C_t_-C_t-1_)	C_t_ (E_t_-E_t-1_)	Total	%
Child's age	−0.007	−0.007	−0.004	−0.010	−0.014	16.66
Child's age squared	0.019	0.006	0.007	0.018	0.025	−29.79
Birth Order	0.002	0.002	−0.001	0.005	0.004	−4.52
Antenatal visit to doctor	0.007	−0.009	0.003	−0.005	−0.002	2.63
Delivery at health facility	0.013	−0.022	0.001	−0.010	−0.009	10.58
Early initiation of breastfeeding	−0.004	0.003	0.002	−0.003	−0.001	0.68
Maternal Schooling (years)	0.038	−0.052	0.010	−0.024	−0.015	17.16
Paternal Schooling (years)	0.003	0.007	0.004	0.005	0.009	−11.13
Maternal CED (Chronic Energy Deficiency)	−0.003	0.000	−0.002	−0.001	−0.003	4.03
Maternal short stature (<145 cm)	−0.007	−0.001	−0.005	−0.002	−0.008	8.98
Wealth Index	−0.013	−0.009	−0.009	−0.013	−0.022	25.82
Residual					−0.050	58.89
Total	0.047	−0.082	0.006	−0.041	−0.085	

Note: Variation 1 (2) uses E_t_ (E_t-1_) and C_t-1_ (C_t_) to weight changes in C and E respectively. See Wagstaff et al. (2003) for details
